# The Influence of Artificial Light at Night on Asthma and Allergy, Mental Health, and Cancer Outcomes: A Systematic Scoping Review Protocol

**DOI:** 10.3390/ijerph19148522

**Published:** 2022-07-12

**Authors:** Andy Deprato, Himasha Rao, Hannah Durrington, Robert Maidstone, Ana Adan, Jose Francisco Navarro, Anna Palomar-Cros, Barbara N. Harding, Prasun Haldar, Saibal Moitra, Tanusree Moitra, Lyle Melenka, Manolis Kogevinas, Paige Lacy, Subhabrata Moitra

**Affiliations:** 1 Alberta Respiratory Centre and Division of Pulmonary Medicine, Department of Medicine, University of Alberta, Edmonton, AB T6G 2R3, Canada; adeprato@ualberta.ca (A.D.); himasha@ualberta.ca (H.R.); placy@ualberta.ca (P.L.); 2 Faculty of Kinesiology, Sport, and Recreation, University of Alberta, Edmonton, AB T6G 2H9, Canada; 3 Department of Biological Sciences, University of Alberta, Edmonton, AB T6G 2E9, Canada; 4 Division of Infection, Immunity, and Respiratory Medicine, School of Biological Sciences, University of Manchester, Manchester M13 9PL, UK; hannah.durrington@manchester.ac.uk; 5 Oxford Centre for Diabetes, Endocrinology and Metabolism, University of Oxford, Oxfordshire OX1 2JD, UK; robert.maidstone@ocdem.ox.ac.uk; 6 Department of Clinical Psychology and Psychobiology, University of Barcelona, 08007 Barcelona, Spain; aadan@ub.edu; 7 Institute of Neurosciences, University of Barcelona, 08007 Barcelona, Spain; 8 Department of Psychobiology and Methodology of Behavioral Sciences, University of Malaga, 29071 Malaga, Spain; navahuma@uma.es; 9 Non-Communicable Diseases and Environment Group, ISGlobal, 08003 Barcelona, Spain; anna.palomar@isglobal.org (A.P.-C.); barbara.harding@isglobal.org (B.N.H.); manolis.kogevinas@isglobal.org (M.K.); 10 Department of Experimental and Health Sciences, University of Pompeu Fabra, 08003 Barcelona, Spain; 11 Department of Physiology, West Bengal State University, Barasat 700126, India; ssprasun0@gmail.com; 12 Department of Medical Laboratory Technology, Supreme Institute of Management and Technology, Mankundu 712139, India; 13 Department of Respiratory Medicine, Apollo Gleneagles Hospital, Kolkata 700054, India; saibal.moitra@gmail.com; 14 Department of Psychology, Barrackpore Rastraguru Surendranath College, Barrackpore 700120, India; moitra.tanusree19@gmail.com; 15 Synergy Respiratory and Cardiac Care, Sherwood Park, AB T8H 0N2, Canada; l.melenka@synergyrespiratory.com; 16 Centro de Investigación Biomédica en Red Epidemiología y Salud Pública (CIBERESP), 08003 Barcelona, Spain

**Keywords:** artificial light at night, asthma, allergies, mental health, cancer

## Abstract

Artificial light at night (ALAN) exposure is associated with the disruption of human circadian processes. Through numerous pathophysiological mechanisms such as melatonin dysregulation, it is hypothesised that ALAN exposure is involved in asthma and allergy, mental illness, and cancer outcomes. There are numerous existing studies considering these relationships; however, a critical appraisal of available evidence on health outcomes has not been completed. Due to the prevalence of ALAN exposure and these outcomes in society, it is critical that current evidence of their association is understood. Therefore, this systematic scoping review will aim to assess the association between ALAN exposure and asthma and allergy, mental health, and cancer outcomes. This systematic scoping review will be conducted in accordance with the Preferred Reporting Items for Systematic reviews and Meta-Analyses statement. We will search bibliographic databases, registries, and references. We will include studies that have described potential sources of ALAN exposure (such as shift work or indoor and outdoor exposure to artificial light); have demonstrated associations with either allergic conditions (including asthma), mental health, or cancer-related outcomes; and are published in English in peer-reviewed journals. We will conduct a comprehensive literature search, title and abstract screening, full-text review, and data collection and analysis for each outcome separately.

## 1. Introduction

Light is the strongest zeitgeber, or synchroniser, for the circadian system [1,2]. Since ancient times, sunlight (or its absence) has remained the most potent environmental cue that regulates human life, activity, and behaviour. However, following the discovery of the electric lamp, modern human processes have been significantly influenced by artificial light. Although artificial light has made our nights more enjoyable and productive, it has also imparted a tremendous adverse effect on the ecosystem at large [3,4]. Light pollution has become a serious environmental concern as artificial light at night (ALAN) severely disrupts the biological cycles of plants, insects, and nocturnal animals by altering their circadian rhythms [5,6,7,8,9,10,11,12]. ALAN exposure comes from many sources, with indoor light being the most prominent. The societal conceptualization of shift work has resulted in humans engaging in work that is dependent to a large degree on artificial light [13]. Moreover, due to the increased use of artificial light, humans generally spend less time in the dark at night.

Most human physiological processes are periodically regulated through circadian timing systems [14]. This internal biological clock (also known as the “Circadian Clock”) functions through circadian pacemaker neurons, particularly those of the hypothalamic suprachiasmatic nucleus (SCN), the master clock of this system [15]. These central neuronal clocks interact with peripheral clocks, which are found in a multitude of tissues throughout the human body [16]. However, ALAN exposure imposes a direct challenge to circadian rhythms as it has been found to disrupt SCN activity and various ensuing mechanisms, particularly by suppressing and dysregulating melatonin release from the pineal gland [17,18,19,20]. As most of our physiological functions are regulated by the cross-talk between the central and peripheral pacemakers, ALAN exposure can consequently alter organ function at different levels by disrupting the synchrony between these pacemakers [21]. Therefore, the increasing prevalence of ALAN exposure in society is important to consider due to its potentially harmful effects on human health.

In this scoping review, we aim to systematically evaluate the current evidence on the association between ALAN exposure and its potential health consequences, particularly in relation to allergic conditions, mental health, and cancer.

## 2. Rationale

There are several ways through which humans can be exposed to ALAN both indoors and outdoors. In addition to exposure in the home or at workplaces (particularly due to shift work), streetlights and light-emitting diodes (LEDs), particularly on the screens of televisions, computers, and mobile phones, are increasingly pervasive sources of ALAN in society. In the past two decades, several epidemiological studies have demonstrated that ALAN exposure has detrimental impacts on sleep and sleeping behaviour [18,20,22,23,24]. While the majority of existing ALAN studies were primarily focused on sleep, some recent studies have demonstrated that ALAN is also associated with other physiological and behavioural functions. However, studies elucidating the role of ALAN exposure on allergic diseases, mental health, and cancer (particularly for subtypes other than breast cancer) are relatively scarce. Therefore, a comprehensive evaluation of current evidence is important to further understand the complex interactions between this environmental perturbation and health.

### 2.1. Allergic Conditions including Asthma

According to recent Global Burden of Disease (GBD) studies, asthma and other atopic diseases such as allergic rhinitis, food allergy, and atopic dermatitis affect approximately 20% of the global population [25,26]. Allergic symptoms and their underlying inflammatory pathways have been observed to vary with the time of day [27,28,29]. Additionally, in asthma, airway diameter is directly controlled by circadian rhythms [30]. Therefore, there is reasonable evidence to suggest that circadian disruption through mechanisms such as ALAN exposure can exacerbate asthma and allergic conditions [14]. For instance, night shift work and evening chronotypes have been associated with worsened asthmatic conditions, suggesting the potential involvement of exposure to ALAN as a perturbation of circadian function [31,32].

### 2.2. Mental Health

Mental health is becoming increasingly important in modern society and reports estimate that 125.3 million individuals worldwide experience disability due to mental disorders [33]. Circadian function has been implicated in psychiatric and neurodegenerative diseases through a number of potential mechanisms [34,35,36,37]. Mental illnesses can be understandably complex, however, there is growing evidence of associations between these conditions and circadian dysfunction as well [38,39,40], which could possibly be mediated by altered sleep behaviour [38]. Additionally, disruption of circadian function through light exposure has long been considered in seasonal behavioural disorders [41]. Although the exact mechanisms of this relationship are not completely understood, the potential association between ALAN exposure and mental health outcomes could be of great use to better understand, with emerging evidence currently being uncovered [24].

### 2.3. Cancer

Cancer is one of the leading causes of morbidity and mortality across the world and results in around 10 million global deaths a year [42,43]. Although cellular and animal studies have shown relationships between clock genes and cancer [44,45,46,47,48], investigations linking circadian disruption and cancer outcomes in humans were not prevalent until the beginning of the 21st century. Early epidemiological studies demonstrated a link between circadian disruption and cancer in the Nurses’ Health Study [49,50]. Considering the greater health risks, particularly of cancer, that have been identified through subsequent work, the International Agency for Research on Cancer (IARC) has recognised night shift work as a potential carcinogen for breast, prostate, and colorectal cancer [51], and workers in Denmark have advocated and received compensation for potentially related breast cancer cases [52]. Several studies have investigated associations between ALAN exposure and cancer outcomes; however, there has not yet been a critical review of existing evidence to understand its association with cancer [53,54,55,56].

Given existing evidence and the importance of the health outcomes defined, it is of great public health interest that a critical analysis of existing research is performed to synthesise available findings on the association between ALAN exposure and asthma and allergy, mental health, and cancer outcomes.

## 3. Objectives

The objective of this systematic scoping review is to assess the associations between ALAN exposure and asthma and allergies, mental health conditions, and cancer-related outcomes. This review will provide a critical overview of current evidence concerning ALAN exposure and a number of chronic health conditions such as asthma and allergy, mental health, and cancer to generate a more comprehensive overview of these topics, which may help in shaping clinical care, diagnosis, management, and public policies.

## 4. Methods and Analysis

### 4.1. Study Design

Scoping reviews allow for greater methodological flexibility and the synthesis of broader topics than systematic reviews [57]. Due to our primary outcomes, we decided to follow a scoping review study design while incorporating components of a systematic review according to the Preferred Reporting Items for Systematic Review and Meta-Analysis Extension for Scoping Reviews (PRISMA-ScR) guidelines [57].

### 4.2. Eligibility Criteria

Studies will be assessed for inclusion in the review according to the following criteria:

Study Design and Study Population: We will only include human studies that investigate the relationship between ALAN exposure and either asthma and allergy, mental health, or cancer outcomes. This includes observational studies including cohort, case-control, and cross-sectional studies if they have (i) clearly demonstrated the effect of exposures that resemble ALAN or similar exposures and (ii) estimated specific outcomes that are relevant to the topics of interest of this review. We will not limit included studies by population demographics or by study size. However, studies with a small number of individuals will be considered in the discussion as necessary. Studies interested in specific populations, such as paediatric or pregnant individuals, will be included and indicated accordingly to assist in the interpretation of results. We will include studies that make comparisons between ALAN exposure and asthma and allergy, mental health, or cancer outcomes. We will consider studies with interventions if encountered. However, due to the topics of interest in our investigation, it is likely that most studies will be observational in design.

Outcomes: We will include studies that investigate the association between ALAN exposure and our review outcomes through measures such as relative risk/risk ratios, prevalence ratios, and odds ratios depending on the study design. Descriptive analyses involving prevalence/incidence rates and incidence rates of review outcomes or measures of ALAN exposure will also be considered.

Additional Characteristics: We will not limit included studies by their ethnicity, country of origin, or geographic region nor by the time frame of their data collection or follow-up. We will limit included studies to those published in English as full texts in peer-reviewed journals. We will not limit included studies by their publication date, but we will be cognisant of those published before the year 2000 due to considerable changes in the technology used for ALAN assessment.

### 4.3. Information Sources

Literature search strategies will be developed for each review outcome using terminology associated with ALAN exposure and either asthma and allergies, mental health, or cancer. We will search the electronic databases MEDLINE (1946 onwards via Ovid), EMBASE (1974 onwards via OVID), PubMed, Scopus, Web of Science, and the Cochrane Database of Systematic Reviews (Cochrane Library). We will also search the registries Cochrane Protocols (Cochrane Library) and the International Prospective Register of Systematic Reviews (PROSPERO) to identify planned, ongoing, or recently published systematic reviews. Additional searches of grey literature will include the first 100 results of a Google Scholar search, hand searches, contact with study authors, and bibliographies from included studies, known reviews, or texts. These searches will be done through the University of Alberta library services. We will continue searches until we attain reasonable confidence that essentially all relevant studies for each review outcome have been considered for inclusion.

### 4.4. Search Strategy

The specific literature search strategies will be developed by one author (AD) and reviewed by the research team. Search strategies will consist of terms relevant to ALAN exposure and either asthma and allergies, mental health, or cancer. Draft MEDLINE search strategies for the three outcomes of interest will include the following terms (Table 1):

The initial search of databases, registries, and Google Scholar will not be limited by study design, year, language, publication status, or other limiters. These study characteristics will be considered as inclusion criteria after the initial search to ensure that studies are not excluded due to improper categorisation within databases. The search strategies will be updated after trial searches are completed, where the first 100 results of these searches will be viewed to ensure that a reasonable proportion of eligible studies are being retrieved. The final search strategies will be reported in the finalised study.

### 4.5. Study Records

Study records will be managed separately for each of the three review outcomes according to the process outlined below:

Data Management: Search results from each database will be uploaded to the systematic review managing software Covidence (Melbourne, VIC, Australia). Covidence will be used for the removal of duplicate study records and the title and abstract screening. Full texts of studies will be retrieved through accessing databases and university librarians in the event of rarity. Results from the full-text review will be recorded in a spreadsheet document. Included studies will be managed and referenced through citation management software.

Selection Process: One reviewer will manage the initial search results by removing duplicates. Review authors will then screen titles and abstracts according to the inclusion criteria. Two authors will then independently complete the full-text review of studies that either met the inclusion criteria or that the reviewers were uncertain of during the title and abstract review with disagreements resolved by consensus or third-party adjudication as needed. The reasons for excluding studies during the full-text review will be recorded. This selection process will be summarised in a PRISMA flow diagram in the finalised study [58].

Data Collection Process: Author pairs will independently collect data from included studies for analysis. Any disagreements concerning collected data items will be resolved by consensus or third-party adjudication as needed. The data collected from eligible studies will include measures of ALAN exposure, review outcomes, and their association. Any process of obtaining additional information on included studies will be done by contacting corresponding authors. Information that is reported in multiple studies will only be included with respect to the original study it was reported in if this is available.

### 4.6. Data Items

We will collect measures of association between ALAN exposure and either asthma and allergy, mental health, or cancer outcomes from included studies such as relative risk/risk ratios, prevalence ratios, and odds ratios. We will also collect descriptive information, for example, prevalence rates and incidence rates of review outcomes and measures of ALAN exposure. We will collect the types of both indoor and outdoor ALAN exposure considered in studies. Demographic information of study populations such as age, sex, height, weight, socioeconomic status, quality of life measures, ethnicity, country of origin, or geographic region will also be collected.

### 4.7. Outcomes and Prioritisation

The primary outcome in this review will be the association between ALAN exposure and review outcomes from included studies, described through relative risk/risk ratios, prevalence ratios, and odds ratios for cases compared to controls. We will also complete a meta-analysis of the associations found between ALAN exposure and cancer outcomes if data allows. Based on the availability of adequate references, we will further implement a meta-regression comparing the highest versus lowest exposure approach as applicable. Additional meta-analyses will be considered if there are enough studies available for the outcome. This includes subgroup and sensitivity analyses to consider differences in the influence of indoor and outdoor ALAN exposure and excluding outlier studies to further explore our review outcomes. If quantitative analysis of outcomes is not feasible, we will still provide a qualitative analysis as appropriate. Secondary outcomes will include descriptive measures of ALAN exposure and outcome rates from included studies to interpret analyses and further inform discussion or conclusions from the review.

### 4.8. Risk of Bias in Individual Studies

Included studies will be assessed for quality by author pairs for each review outcome. The quality assessment tool will be based on the study design. We will assess cohort and case-control study designs through the respective Newcastle-Ottawa Scale (NOS) [59]. We will assess cross-sectional studies through the Appraisal Tool for Cross-Sectional Studies (AXIS) [60]. If encountered, we will assess randomised control trials through the revised Cochrane risk of bias tool for randomised trials (RoB2) [61], and non-randomised experimental studies through the Risk of Bias in Non-randomised Studies—of Interventions (ROBINS-I) tool [62]. Quality assessment results for each of the three review outcomes will be reported in individual tables.

### 4.9. Data Synthesis

We will provide descriptive statistics and summary tables of the demographic characteristics and review outcomes of interest reported for each study. We will consider completing quantitative data analysis and synthesis for each review outcome when appropriate and present these results through corresponding tables and figures. This includes meta-analysis, meta-regression, and highest versus lowest exposure approaches. We will also consider completing further subgroup and sensitivity analyses for each review outcome. If adequate references are available, we will perform a stratification analysis by considering indoor and outdoor types of ALAN exposure as subgroups. Any specific statistical software and methods used for relevant analyses will be reported in the finalised review. When quantitative synthesis for a review outcome is not appropriate, we will still include a narrative synthesis of findings.

### 4.10. Meta-Bias(es)

We will assess the potential for publication bias through funnel plots for each review outcome if ≥6 studies are available for that outcome as per previous reports [63]. The magnitude of small-study effects will be assessed according to recent methodologies where applicable [64]. Funnel plots for eligible review outcomes will be included in the finalised study with a qualitative statement of any potential publication biases detected. Statements will also be made concerning small-study effects given the results of this assessment. Furthermore, any influence of probable artifacts, such as publications with negative results, extreme values, or articles published in predatory journals will be thoroughly scrutinized.

### 4.11. Confidence in Cumulative Evidence

The Grading of Recommendations, Assessment, Development, and Evaluations (GRADE) methodology will be applied to the body of evidence for each of the three outcomes included in this review [65]. The average quality of evidence will be assessed across all GRADE domains as high, moderate, low, or very low for each outcome to help guide the discussion of the strengths and limitations of included studies and confidence in conclusions drawn from analyses. Quality of evidence assessment results for each of the three review outcomes will be reported in individual tables.

## 5. Study Timeline

**Figure 1:**
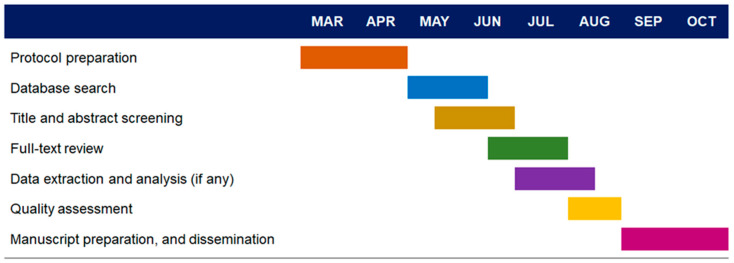
Gantt chart describing the proposed timeline for this review including each of the three outcomes of interest. Months are for the year 2022.

The proposed timeline for this review is included below (Figure 1). The dates included refer to the expected completion date for the item described. This timeline will apply to each of the three study outcomes as they are worked on concurrently by the different centres involved in this review.

## 6. Conclusions

In this scoping review, we expect to generate high-quality evidence of ALAN-associated health effects, particularly on allergic conditions, mental health, and cancer. This will serve as a composite and up-to-date analysis of observational studies performed across the world delineating an emerging public and planetary health concern.

## Figures and Tables

**Table 1:** Draft MEDLINE search strategies to identify studies for inclusion for the three review outcomes of interest.

Interested Outcome	Example MEDLINE Search Strategy
Asthma and Allergies	Ovid MEDLINE(R) ALL <1946 to 16 June 2022> 1. (artificial light at night or ALAN or light at night).mp. 2. (blue light * or white light * or artificial light *).mp. 3. (night * or dark * or sleep * or chronotyp *).mp. 4. (asthma * or allerg * or immun *).mp. 5. 2 and 3 6. 1 or 5 7. 4 and 6 mp = title, abstract, original title, name of substance word, subject heading word, floating sub-heading word, keyword heading word, organism supplementary concept word, protocol supplementary concept word, rare disease supplementary concept word, unique identifier, synonyms 379 results returned
Mental Health	Ovid MEDLINE(R) ALL <1946 to 16 June 2022> 1. (artificial light at night or ALAN or light at night).mp. 2. (blue light * or white light * or artificial light *).mp. 3. (night * or dark * or sleep * or chronotyp *).mp. 4. (mental health or mental illness or anxiety or depression or mood or schizophrenia or bipolar disorder or sleep disorder or insomnia).mp. 5. 2 and 3 6. 1 or 5 7. 4 and 6 mp = title, abstract, original title, name of substance word, subject heading word, floating sub-heading word, keyword heading word, organism supplementary concept word, protocol supplementary concept word, rare disease supplementary concept word, unique identifier, synonyms 341 results returned
Cancer	Ovid MEDLINE(R) ALL <1946 to 16 June 2022> 1. (artificial light at night or ALAN or light at night).mp. 2. (blue light * or white light * or artificial light *).mp. 3. (night * or dark * or sleep * or chronotyp *).mp. 4. Cancer *.mp.a 5. 2 and 3 6. 1 or 5 7. 4 and 6 mp = title, abstract, original title, name of substance word, subject heading word, floating sub-heading word, keyword heading word, organism supplementary concept word, protocol supplementary concept word, rare disease supplementary concept word, unique identifier, synonyms 429 results returned

## Data Availability

This protocol has been adapted from the Preferred Reporting Items for Systematic Review and Meta-Analysis Protocols statement. These guidelines were used for direction in developing this protocol as no formalised resources for scoping review or systematic scoping review protocols are available at this time.
